# Fenretinide mediated retinoic acid receptor signalling and inhibition of ceramide biosynthesis regulates adipogenesis, lipid accumulation, mitochondrial function and nutrient stress signalling in adipocytes and adipose tissue

**DOI:** 10.1016/j.bcp.2015.11.017

**Published:** 2016-01-15

**Authors:** George D. Mcilroy, Seshu R. Tammireddy, Benjamin H. Maskrey, Louise Grant, Mary K. Doherty, David G. Watson, Mirela Delibegović, Phillip D. Whitfield, Nimesh Mody

**Affiliations:** a Institute of Medical Sciences, College of Life Sciences & Medicine, University of Aberdeen, Aberdeen, UK; b Lipidomics Research Facility, Department of Diabetes and Cardiovascular Science, University of the Highlands and Islands, Inverness, UK; c Metabolomics Group, Strathclyde Institute of Pharmacy and Biomedical Sciences, University of Strathclyde, Glasgow, UK

**Keywords:** Fenretinide, Retinoic acid, Adipocytes, Metabolomics, Dihydroceramide, Lipidomics

## Abstract

Fenretinide (FEN) is a synthetic retinoid that inhibits obesity and insulin resistance in high-fat diet (HFD)-fed mice and completely prevents 3T3-L1 pre-adipocyte differentiation. The aim of this study was to determine the mechanism(s) of FEN action in 3T3-L1 adipocytes and in mice. We used the 3T3-L1 model of adipogenesis, fully differentiated 3T3-L1 adipocytes and adipose tissue from HFD-induced obese mice to investigate the mechanisms of FEN action. We measured expression of adipogenic and retinoid genes by qPCR and activation of nutrient-signalling pathways by western blotting. Global lipid and metabolite analysis was performed and specific ceramide lipid species measured by liquid chromatography-mass spectrometry. We provide direct evidence that FEN inhibits 3T3-L1 adipogenesis *via* RA-receptor (RAR)-dependent signaling. However, RARα antagonism did not prevent FEN-induced decreases in lipid levels in mature 3T3-L1 adipocytes, suggesting an RAR-independent mechanism. Lipidomics analysis revealed that FEN increased dihydroceramide lipid species 5- to 16-fold in adipocytes, indicating an inhibition of the final step of ceramide biosynthesis. A similar blockade in adipose tissue from FEN-treated obese mice was associated with a complete normalisation of impaired mitochondrial β-oxidation and tricarboxylic acid cycle flux. The FEN catabolite, 4-oxo-*N*-(4-hydroxyphenyl)retinamide (4-OXO), also decreased lipid accumulation without affecting adipogenesis. FEN and 4-OXO (but not RA) treatment additionally led to the activation of p38-MAPK, peIF2α and autophagy markers in adipocytes. Overall our data reveals FEN utilises both RAR-dependent and -independent pathways to regulate adipocyte biology, both of which may be required for FEN to prevent obesity and insulin resistance *in vivo*.

## Graphical abstract

Proposed mechanistic action of FEN in adipocytes. RA inhibits adipogenesis through early RAR activation, which PPARγ agonist ROSI cannot prevent. FEN inhibits adipogenesis with a delayed response in RAR signalling, however, inhibition is lost when combined with ROSI. The 4-OXO FEN catabolite cannot inhibit adipogenesis through RAR activation, but like FEN’s RAR-independent effects, displays increased phosphorylation of Akt, mild cellular stress/autophagy induction and decreased lipid accumulation. Moreover, FEN-mediated inhibition of DES-1 increases dihydroceramide levels in a RAR-independent manner, and is linked to a complete prevention of mitochondrial dysfunction and decreased adiposity in high-fat fed obese mice. Thus an alternative to the additive beneficial effects of FEN-mediated RAR-dependant and -independent signalling, 4-OXO FEN may be a novel therapeutic candidate to improve adipocyte hypertrophy *via* inhibition of ceramide biosynthesis and modulation of nutrient stress pathways.

**Figure:**
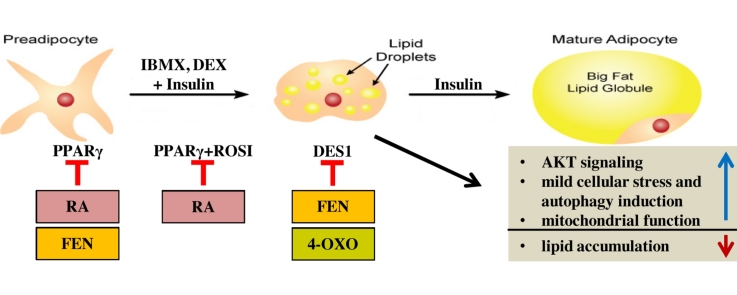

## 1 Introduction

Retinol (vitamin A) and the retinoid metabolism pathway play an important role in body mass regulation and adipocyte biology [1], [2], [3], [4], [5], [6]. Targeting retinoid homeostasis may therefore offer a therapeutic approach for obesity and type-2 diabetes. Vitamin A is a lipid soluble molecule which undergoes multiple steps of metabolism through a complex pathway of enzymes and transport proteins [7]. All-*trans*-retinoic acid (RA), which is the most active metabolite of retinol, has long been known to inhibit adipogenesis through the prevention of C/EBPβ mediated transcription [1], [8], More recently, RA has been shown to improve obesity and glucose homeostasis *in vivo* [4]. However, with prolonged exposure, naturally derived retinoid compounds such as RA and retinyl-acetate can lead to liver toxicity, which restricts their potential use as therapeutic agents [9].

Fenretinide (FEN), otherwise known as *N*-(4-hydroxyphenyl)retinamide or 4-HPR, is a structural derivative of RA with reduced toxicological profile [10], [11]. FEN treatment leads to increased renal clearance and thus decreased serum levels of the retinol transport protein, serum retinol binding protein (gene name *Rbp4*) in both humans and mice [12], [13], [14], [15], [16]. This decrease in circulating RBP4 levels had been proposed to be the mechanism that FEN treatment led to prevention of insulin resistance associated with high-fat diet (HFD) induced obesity [15]. However, FEN has also been shown to reduce obesity and hyperleptinaemia in mice lacking RBP4, implying that the anti-obesity effects of FEN are most likely to be independent of its ability to decrease circulating RBP4 levels [16].

Thus, the beneficial effects of FEN appear to be through several different mechanisms including alterations to retinoid homeostasis in multiple tissues [6], increased hepatic lipid oxidation [17] and inhibition of ceramide biosynthesis leading to an increase in dihydroceramide in muscle and liver [18]. Studies in cancer cells have found that both FEN and dihydroceramide treatment are associated with the activation of cellular stress responses and autophagy induction [19], [20], [21]. Autophagy plays a crucial role in cellular homeostasis through the degradation and recycling of organelles such as mitochondria or ER and the regulation of intracellular lipid stores [22], [23]. Since defective autophagy may also underlie impaired insulin sensitivity in obesity and upregulating autophagy may be a useful strategy to combat insulin resistance [24], [25], [26], it highlights the importance to further characterise the biological effects of FEN.

In addition to the beneficial effect of FEN *in vivo*, we recently demonstrated that FEN (similarly to RA) is able to inhibit 3T3-L1 adipocyte differentiation by blocking transcription of C/EBPα and PPARγ, master regulators that synergistically coordinate adipogenesis and adipocyte biology and simultaneously increasing retinoid gene expression [6]. Interestingly, rosiglitazone (ROSI), a thiazolidinedione and PPARγ agonist commonly used to stimulate adipogenesis, suppressed gene expression of all the retinoid metabolism markers examined in differentiating 3T3-L1 cells [6]. FEN also decreased lipid accumulation in fully differentiated 3T3-L1 adipocytes [6], but RA treatment did not [27], [28]. Thus, FEN and RA appear to have divergent biological effects which may be due to unique activation of retinoic acid receptor (RAR)-dependent and -independent pathways. Since, it is unknown which pathway FEN requires for its biological effects in adipocytes we aimed to determine the mechanism(s) of FEN action in the 3T3-L1 model of pre-adipocyte differentiation and fully differentiated adipocytes. In addition, we have translated some of our new findings to adipose tissue of HFD-induced obese mice.

## 2 Methods

### 2.1 Cell culture

3T3-L1 pre-adipocytes were maintained and differentiated for 8 days (or 16 days where stated) as previously described [6]. C3H10T1/2 cells were similarly treated however penicillin/streptomycin was omitted from the media. DMSO was used as vehicle control (VEH) and to dissolve all experimental compounds. FEN (Cilag AG, Schaffhausen, Switzerland), RA (Sigma–Aldrich, UK) and ROSI (Cayman Chemical, MI, USA) were used at 1 μM, 4-OXO (Santa Cruz, TX, USA) at 0.5 μM and ER50891 (Tocris Bioscience, Bristol, UK) at 10 μM and added at day 0 (or day 8 where indicated). Cells were stained for neutral lipids with Oil Red O, images taken and then the stain was eluted and quantified at 520 nm.

### 2.2 Lipolysis assay

Basal glycerol levels were measured in media collected at day 16 of differentiation from 3T3-L1 cultures. The Triglyceride Liquid assay (Sentinel Diagnostics #17628) was used following the supplied protocol.

### 2.3 Gene expression and protein analysis

RNA isolation, cDNA synthesis and qPCR were performed at day 8 of differentiation (or as stated in the figure legends) as previously described [6]. Control reactions for contaminating DNA were performed routinely and relative expression calculated using the Pfaffl method [29]. The geometric mean of three stable reference genes (*Nono*, *Ywhaz* and *Actb* or as stated in the figure legend) were obtained from five commonly used sequences and used for normalisation. Primer sequences available on request, some of which were obtained from PrimerBank [30].

SDS-PAGE was performed and transferred to nitrocellulose membranes as described previously [31]. Antibodies against p-eIF2α (#9721), eIF2 α (#5324S), p-p38 MAPK (#9211), p38 MAPK (#8690S), Beclin1 (#3495), LC3B (#3868), p-Akt Ser473 (#9271) were from Cell Signalling, SH-PTP2 (sc-280) and Akt1/2/3 (sc-8312) from Santa Cruz. All antibodies were detected with goat anti-rabbit HRP secondary antibody (#28177) from Anaspec. Proteins were visualized using enhanced chemiluminescence (ECL) and quantified by densitometry scanning using the Fusion imaging system and Bio-1D software (Peqlab).

### 2.4 Global lipidomics analysis of adipocytes

Extraction of 3T3-L1 adipocyte lipids was performed according to the method described by Folch et al. [32]. The lipids were analysed by liquid chromatography–mass spectrometry (LC–MS) using a Thermo Orbitrap Exactive mass spectrometer (Thermo Scientific, Hemel Hempstead, UK), equipped with a heated electrospray ionization (HESI) probe and coupled to a Thermo Accela 1250 UHPLC system. All samples were analysed in both positive and negative ion mode over the mass to charge (*m*/*z*) range 200–2000. The samples were injected on to a Thermo Hypersil Gold C18 column (2.1 mm × 100 mm, 1.9 μm). Mobile phase A consisted of water containing 10 mM ammonium formate and 0.1% (v/v) formic acid. Mobile phase B consisted of 90:10 isopropanol/acetonitrile containing 10 mM ammonium formate and 0.1% (v/v) formic acid. The initial conditions for analysis were 65%A/35%B. The percentage of mobile phase B was increased to 100% over 10 minutes and held for 7 min before re-equilibration with the starting conditions for 4 min. All solvents were LC–MS grade (Fisher Scientific, Loughborough, UK). The raw LC–MS data were processed with Progenesis CoMet v2.0 software (Non-linear Dynamics, Newcastle, UK) and searched against LIPID MAPS (www.lipidmaps.org) for identification.

### 2.5 Animals

Male C57BL/6 mice were randomised by body weight at three months of age and fed CHOW, HFD or FEN-HFD for 20 weeks. Analysed tissues were collected during previously performed experiments as described in [6], whereby FEN-HFD prevented obesity and factors associated with insulin resistance. Perigonadal white adipose tissue (PG-WAT) from *ad libitum* fed mice was rapidly dissected, frozen in liquid nitrogen, and stored at −80 °C. Animal procedures were approved by the University of Aberdeen Ethics Review Board and performed under license (PPL60/3951) approved by the UK Home Office.

### 2.6 Quantitative analysis of ceramides and dihydroceramides in adipose tissue

Lipids were extracted from murine adipose tissue according to the method of Bligh and Dyer [33]. The ceramides and dihydroceramides were then isolated by silica solid phase extraction chromatography. C17:0 ceramide and C12:0 dihydroceramide (Avanti Polar Lipids, Alabaster, AL, USA) were included in the experimental system as internal standards (ISTD). LC–MS/MS analyses were performed in positive ion mode on a Thermo TSQ Quantum Ultra triple quadrupole mass spectrometer equipped with a HESI probe and coupled to a Thermo Accela 1250 UHPLC system. The ceramides and dihydroceramides were separated on a Kinetex 2.6 μm C8 column (100 × 2.1 mm) (Phenomenex, Macclesfield, UK). Mobile phase A consisted of 90% H_2_O, 10% acetonitrile with 0.1% formic acid and mobile phase B consisted of acetonitrile with 0.1% formic acid. The gradient was held at 80% B for 1 min initially, increased to 100% B at 15 min, held at 100% B for 1 min and then re-equilibrated to starting conditions with a total run time of 20 min. The flow rate was 500 μl/min with a column temperature of 40 °C. All solvents were HPLC grade or above (Fisher Scientific, Loughborough, UK). The data were acquired and processed using Xcalibur software v2.1 (Thermo Scientific). The concentration of the ceramide and dihydroceramide molecular species was determined by comparison to calibration curves generated with C16:0 and C24:1 standards (Avanti Polar Lipids, Alabaster, AL, USA). Total ceramide and dihydroceramide concentrations were calculated from the summed concentrations of all the monitored molecular species. All values were normalised to wet weight of PG-WAT.

### 2.7 Metabolomic profiling of adipose tissue

Metabolomic profiling was carried out on a ZICpHILIC column (150 × 4.6 mm, 5 μm, HiChrom, Reading, UK) and an Orbitrap Exactive MS using conditions described previously [34]. Data extraction and data base searching were also carried out as described previously [34].

### 2.8 Statistics

Data represents the mean ± SD and *n* indicates the number of biological replicates. Data were analysed using one-way ANOVA with Tukey’s multiple-comparison post-hoc test (or unpaired Student’s *t*-test where stated) with *P* < 0.05 considered significant.

## 3 Results

### 3.1 PPARγ agonist driven adipocyte differentiation is inhibited by RA but not FEN

**Fig. 1:**
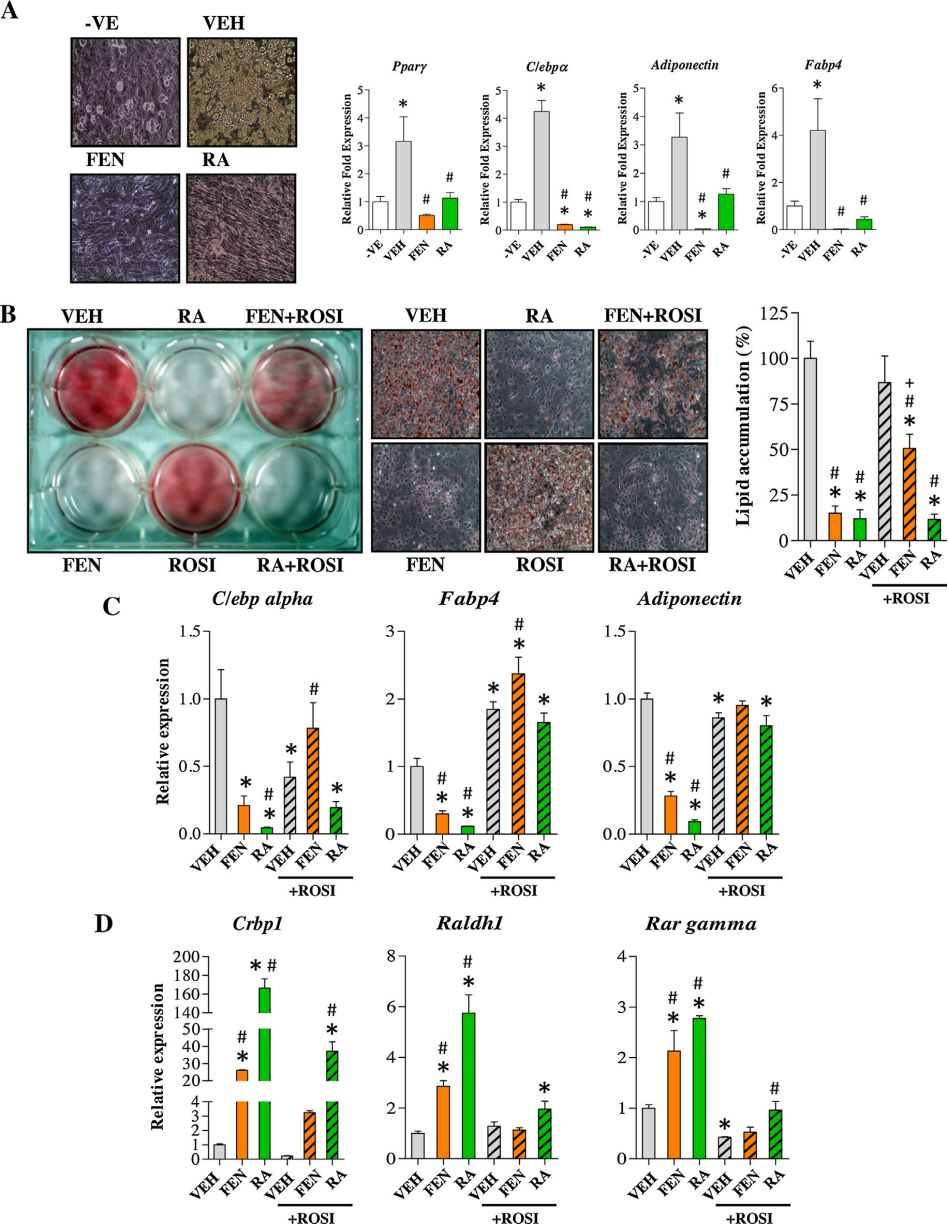
FEN cannot inhibit PPARγ agonist mediated 3T3-L1 differentiation. (A) Lipid stained images at 200× magnification and expression analysis of adipogenic markers of C3H10T1/2 cells differentiated with indicated compounds. Data was normalised to *Nono*, *Ywhaz* and *18S*, *n* = 4 biological replicates. Significance *P < 0.05 vs -VE or #*P* < 0.05 vs VEH by one-way ANOVA with Tukey post hoc test. (B) Lipid stained 3T3-L1 adipocytes, differentiated with indicated compounds. Middle panels are 200× images representative of cell morphology in left panel. Right panel is quantification of lipids, *n* = 5. Significance *P < 0.001 vs VEH, #*P* < 0.001 vs ROSI and +*P* < 0.001 vs FEN. Gene expression analysis of adipogenic (C) and retinoid (D) markers, *n* = 3. Significance *P < 0.05 vs VEH or #*P* < 0.05 vs ROSI.

FEN completely inhibited adipocyte differentiation in the 3T3-L1 cell line [6] and in C3H10T1/2 pluripotent stem cells indicating that inhibition of adipogenesis is a common characteristic of FEN treatment (Fig. 1a). We examined whether FEN could inhibit adipogenesis in 3T3-L1 cells stimulated with ROSI. RA was able to completely inhibit differentiation (Fig. 1b) with ROSI present (RA + ROSI). FEN was not able to inhibit adipogenesis in the presence of ROSI, but decreased lipid accumulation when compared to VEH or ROSI treatments (Fig. 1b). Moreover, RA + ROSI was able to suppress levels of *C/ebp alpha* but FEN + ROSI was unable to replicate this suppression (Fig. 1c). FEN + ROSI could not suppress C/EBPα-PPARγ target genes, *Fabp4*, *Adiponectin* (Fig. 1c) and *Fabp5* (not shown). While RA treatment inhibited adipogenesis in the presence of ROSI in terms of lipid accumulation and *C/ebp alpha* induction, gene expression of terminal markers of adipogenesis (*Fabp4* and *Adiponectin*) appeared unaffected in contrast to treatment with RA alone. These findings highlight that several markers should be used to draw conclusions when assessing adipocyte differentiation.

RA + ROSI induced retinoid homeostasis genes *Crbp1*, *Raldh1* and *Rar gamma*, although the increases in expression were partially suppressed by ROSI (Fig. 1d). FEN + ROSI did not induce these retinoid genes. These results suggest that induction of RA-responsive genes is required for retinoid-mediated inhibition of adipogenesis since PPARγ stimulation can block this inhibition.

### 3.2 RARα antagonism leads to loss of FEN effects on inhibition of adipogenesis

**Fig. 2:**
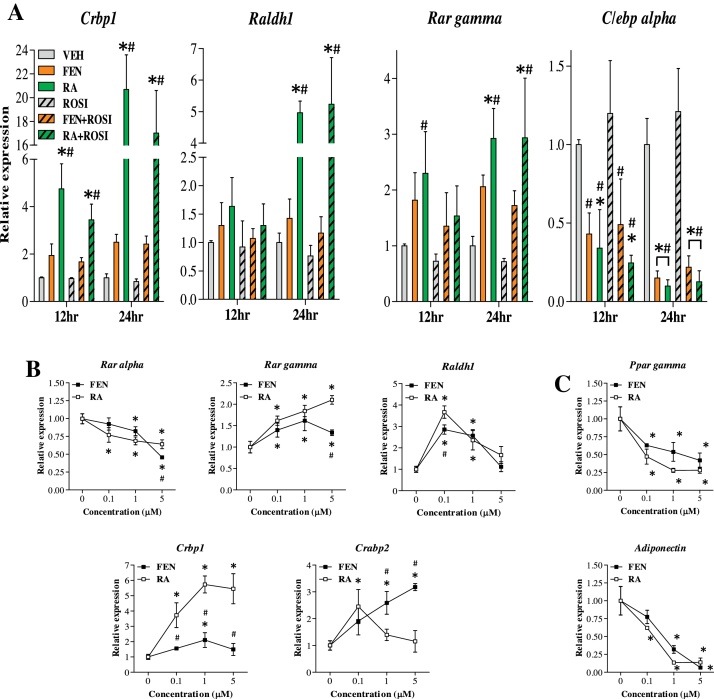
Time and dose-dependent alterations in gene expression between FEN and RA treatment. (A) Gene expression analysis of retinoid and adipogenic markers in 3T3-L1 cells after 12 and 24 h of exposure to MDI and indicated compounds. Data was normalised *Nono*, *Ywhaz* and *Hprt*, *n* = 3 biological replicates. Significance *P < 0.05 vs VEH or #*P* < 0.05 vs ROSI. Expression analysis of retinoid (B) and adipogenic (C) markers in 3T3-L1 adipocytes differentiated for 48 hours with MDI and indicated compounds. Data was normalised to *Nono*, *Ywhaz* and *18S*, *n* = 3 biological replicates. Significance *P < 0.05 vs VEH or #*P* < 0.05 vs RA (at equivalent dose).

**Fig. 3:**
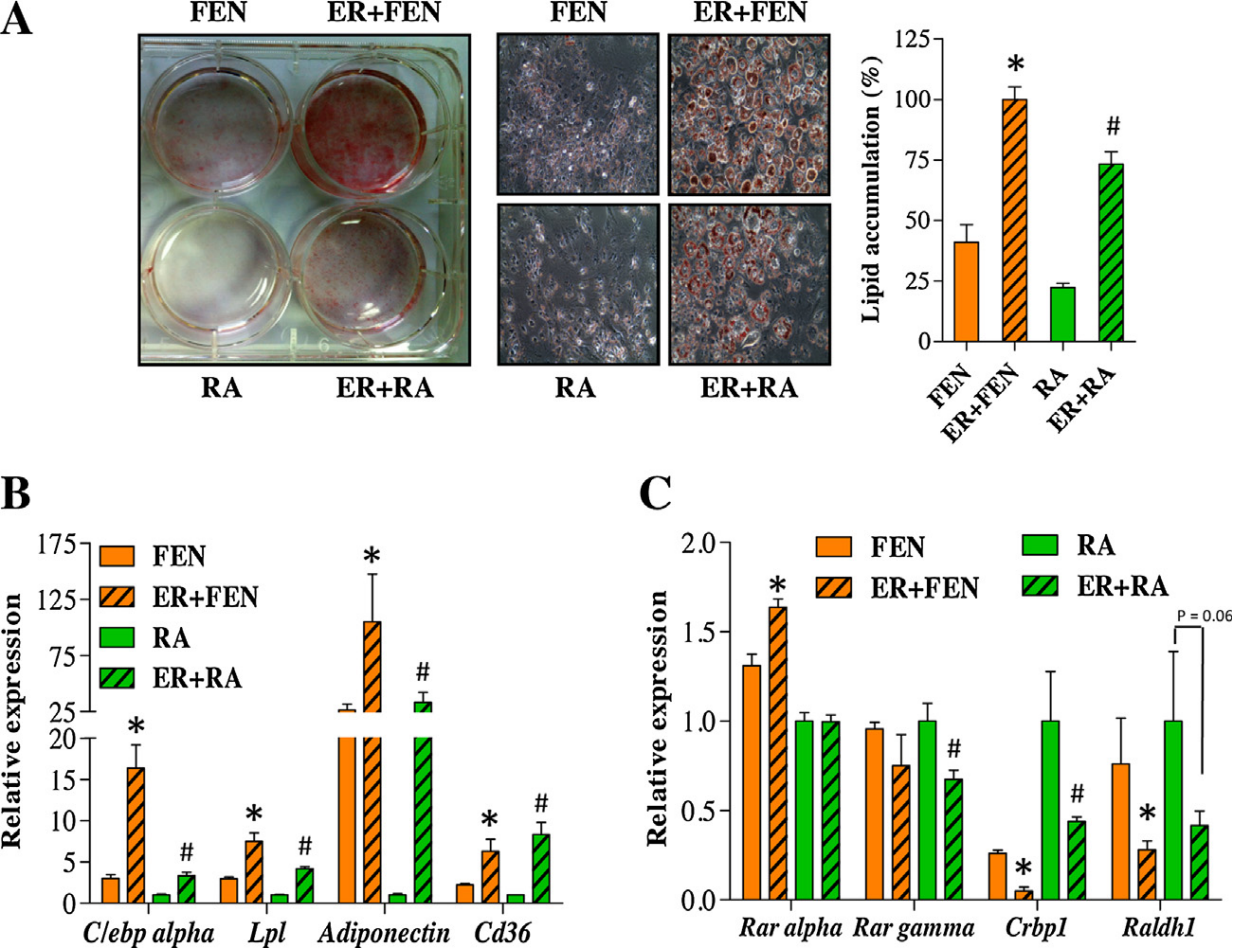
RARα antagonism blocks inhibition of adipogenesis by FEN. (A) Lipid stained 3T3-L1 adipocytes, differentiated with indicated compounds +/− ER50891 (ER). Middle panels are 200× images of cell morphology in left panel. Right panel indicates quantification of lipids, *n* = 6. Significance *P < 0.0001 vs FEN and #*P* < 0.0001 vs RA by *t*-test. Gene expression analysis of adipogenic (B) and retinoid (C) markers. Significance *P < 0.05 vs FEN and #*P* < 0.05 vs RA by *t*-test, *n* = 3.

To account for the differences between FEN and RA observed during ROSI induced differentiation, we hypothesized that FEN induced retinoid signalling is less potent compared to RA. To test this, we measured retinoid-responsive gene expression following FEN/RA treatment during the early stages of adipogenesis (Fig. 2a) and in response to increasing concentrations (Fig. 2b and c). We determined that FEN induced retinoid signalling is 2–3 times less potent than RA. Therefore, next we tested this RAR-dependence by blocking RAR ligand-mediated signalling with a selective RARα antagonist (ER50891). ER50891 blocked RA-induced inhibition of adipogenesis and allowed accumulation of lipid (Fig. 3a). Similarly, ER50891 blocked FEN-induced inhibition of adipogenesis (Fig. 3a). In addition, RARα antagonism prevented inhibition of adipocyte genes (Fig. 3b) and inhibited *Crbp1* and *Raldh1* by ≥50% (Fig. 3c). Overall, these findings strongly demonstrate that the mechanism of FEN action to inhibit 3T3-L1 adipogenesis is mediated by ligand-induced activation of RARα signalling and genes involved in retinoid metabolism.

### 3.3 FEN acts through RAR-independent mechanisms to decrease lipid levels in mature adipocytes

**Fig. 4:**
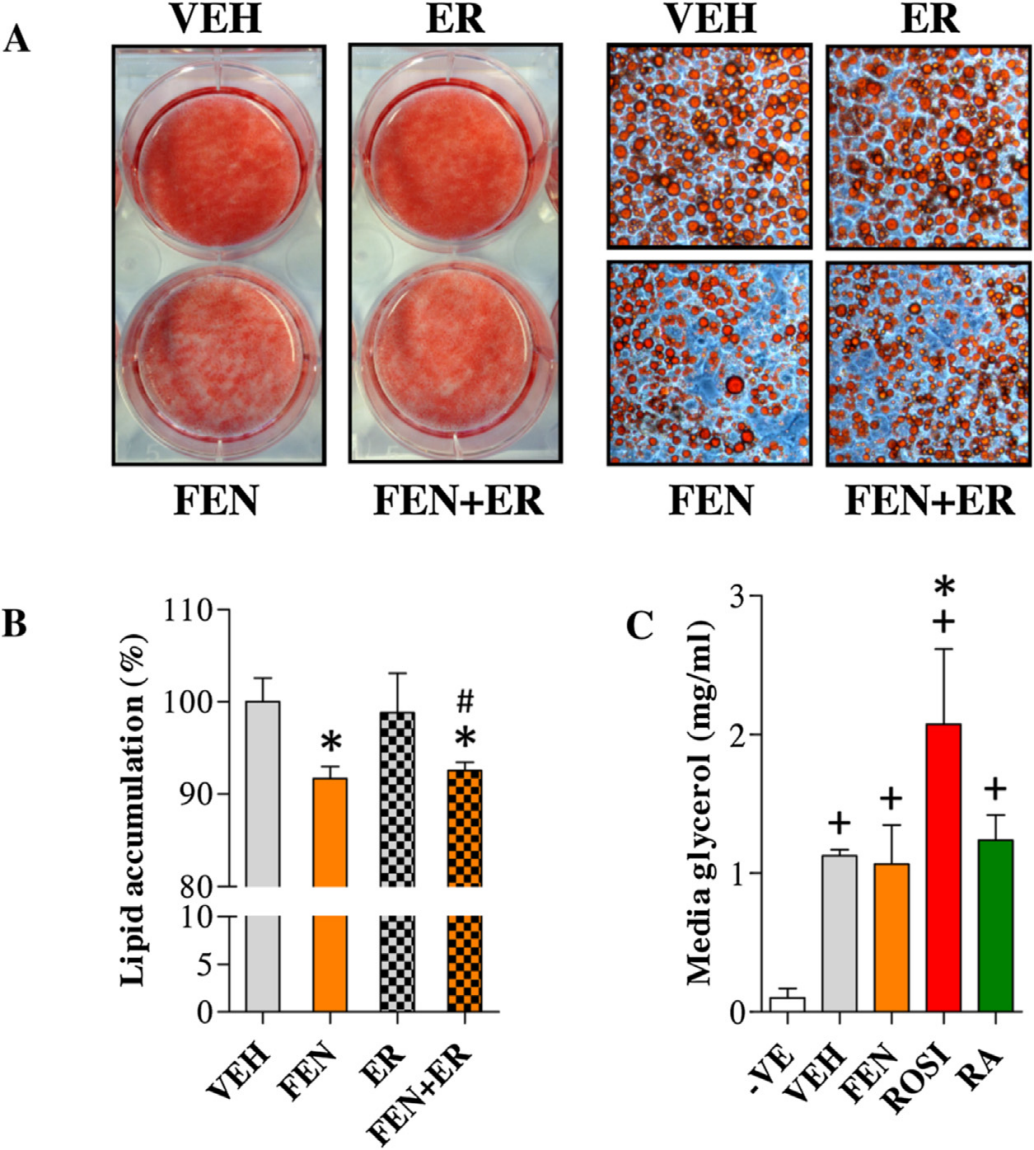
FEN decreases lipid accumulation in mature 3T3-L1 adipocytes independently of RARα signalling. (A) Lipid stained 3T3-L1 adipocytes, differentiated for 16 days with indicated compounds added at day 8. Right panels are 200× images of cell morphology from left panels. (B) Lipid quantification of cultures shown in A, *n* = 6. Significance *P < 0.001 vs VEH and #*P* < 0.01 vs VEH + ER. (C) Basal lipolysis by quantification of glycerol in the culture media from indicated treatments. 3T3-L1 adipocytes were treated as in (A), *n* = 7. Significance +*P* < 0.001 vs -VE and *P < 0.001 vs VEH.

Next, we examined whether RAR-dependent pathways were responsible for the FEN-induced decrease in lipid accumulation in mature 3T3-L1 adipocytes [6]. Antagonism of RARα with ER50891 failed to prevent this effect of FEN (Fig. 4a and b). Moreover, FEN did not affect media glycerol levels when compared to VEH controls suggesting that the basal rate of lipolysis is not altered by FEN (Fig. 4c). ROSI increased media glycerol levels indicating a putative increase in lipolysis consistent with an increase in ATGL expression [6], [35], [36]. These data suggest that the FEN-induced decrease in lipid accumulation in mature 3T3-L1 adipocytes is *via* an RAR-independent mechanism(s).

### 3.4 Lipidomics analysis of FEN treated 3T3-L1 adipocyte cells

Previous studies have identified alterations in ceramide biosynthesis and autophagy induction associated with FEN treatment [19], [37], however it is unknown if FEN can alter these pathways in adipocytes. We performed global lipidomics analysis in mature adipocytes to determine whether ceramide lipid species (*i.e.* sphingolipids) were altered. FEN specifically led to a 9 to 16-fold increase in dihydroceramide (Cer 40:0 and 42:0) compared to VEH and a 5-fold increase in dihydroceramide (Cer 34:0) compared to RA (Table 1). FEN also increased dihydroceramide-containing sphingomyelin (SM 33:0 and 34:0) compared to RA or VEH. FEN treatment led to a reciprocal decrease in ceramide-containing sphingomyelin (SM 42:3 and 34:1) compared to VEH and RA, respectively (Table 1).

Interestingly, both FEN and RA exposure resulted in a complex remodelling of triacylglyceride species, with similar increases (from 2- to 18-fold) in longer chain and desaturated fatty acids (Table 2). FEN and/or RA also decreased a number of triacylglyceride species (Table 1, Table 2). However, FEN decreased many more triacylglyceride lipid species (from 2 to 11-fold) and by a greater amount compared to RA. These findings are in agreement with a FEN-specific (RAR-independent mechanism) to decrease lipid accumulation in mature adipocytes (Fig. 4 and Ref. [6]).

### 3.5 FEN increases adipose dihydroceramide levels *in vivo*

**Fig. 5:**
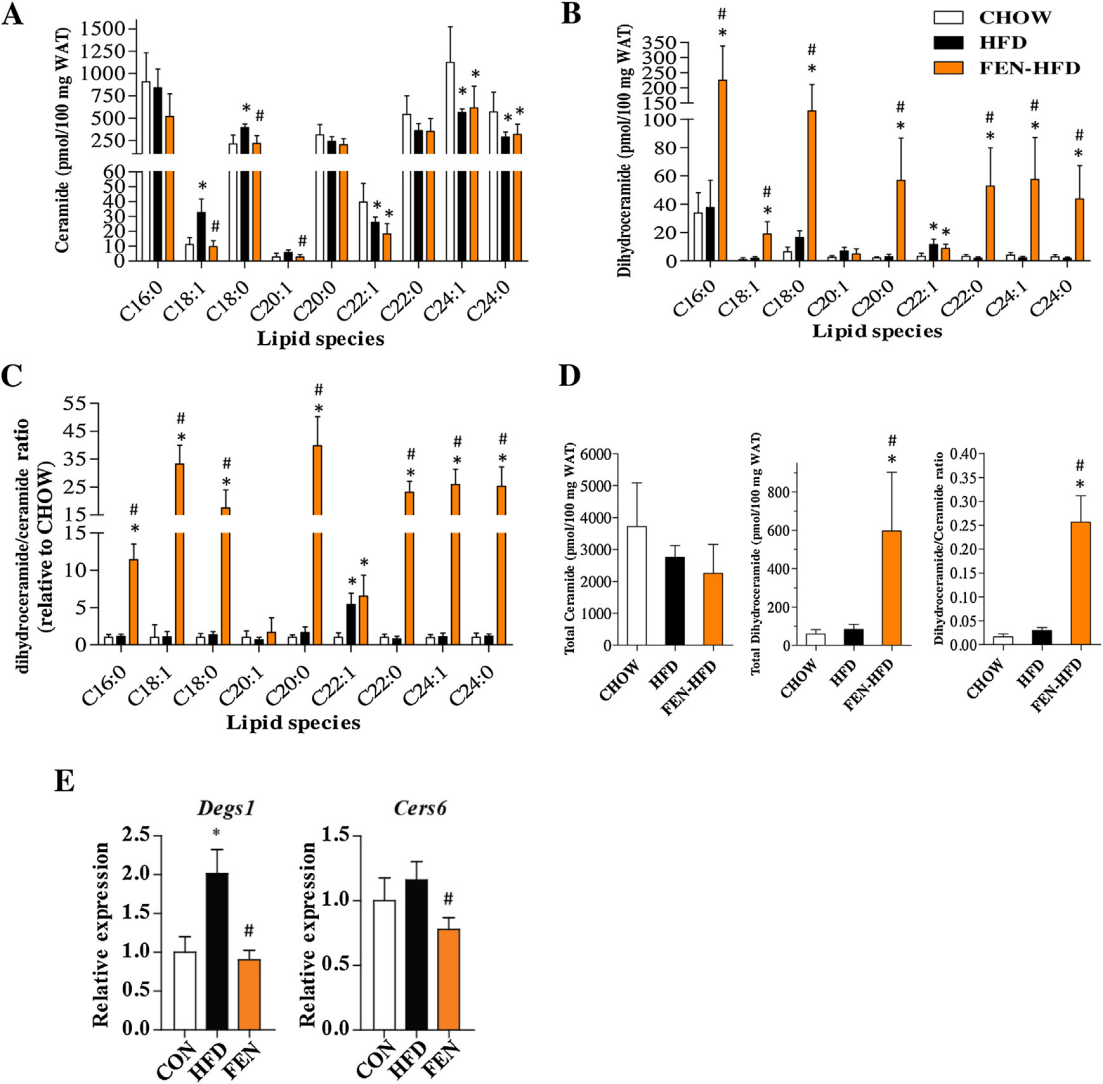
FEN increases dihydroceramide levels in adipose tissue. Quantification of ceramide (A) or dihydroceramide species (B) in PG-WAT from mice fed CHOW, HFD or FEN-HFD. (C) The relative fold change in dihydroceramide to ceramide. (D) Total levels of ceramide, dihydroceramide and the dihydroceramide/ceramide ratio. Significance *P < 0.05 vs CHOW and #*P* < 0.05 vs HFD. *n* = 5 for CHOW, *n* = 6 for HFD and FEN-HFD. (E) Gene expression analysis of WAT from mice fed CHOW (*n* = 6), HFD (*n* = 6) or FEN-HFD (*n* = 8). Data was normalised to *Gapdh*. Significance *P < 0.05 vs CHOW and #*P* < 0.05 vs HFD (by *t*-test for *Cers6*).

To translate our 3T3-L1 adipocyte findings to an *in vivo* setting, we quantified ceramide and dihydroceramide lipid species from PG-WAT of mice fed HFD +/− FEN or normal CHOW for 20 weeks [6]. FEN-HFD prevented obesity, hyperglycemia, insulin resistance and hepatic steatosis in these mice [6]. HFD increased adipose ceramide C18:1 and C18:0 compared to CHOW (Fig. 5a). FEN-HFD completely prevented this elevation. HFD and FEN-HFD both decreased other ceramide species (C22:1, C24:1 and C24:0). Strikingly, FEN-HFD increased almost all dihydroceramides and the ratio of dihydroceramide:ceramide in PG-WAT of treated mice (Fig. 5b and c). Moreover, FEN increased total tissue dihydroceramide levels by 7-fold (Fig. 5d). Enzymes involved in ceramide biosynthesis, dihydroceramide desaturase, (Des1) and ceramide synthase (CerS)-6 have been recently implicated with increased enhanced ceramide production mediating high-fat diet induced metabolic dysregulation in adipose tissue of mice and humans [38], [39], [40]. HFD increased WAT *Des1* gene expression compared to CHOW (Fig. 5e). FEN-HFD completely prevented this elevation; moreover, FEN-HFD decreased *CerS6* gene expression compared to HFD. Therefore, inhibiting adipose ceramide synthesis may contribute to the beneficial effects with respect to adiposity and adipocyte insulin sensitivity previously observed with FEN treatment in mice [6], [16].

### 3.6 FEN completely normalises HFD induced impairments to mitochondrial function *in vivo*

Since excess ceramide synthesis has been negatively associated with mitochondrial function and insulin sensitivity, we performed global metabolomics analysis of PG-WAT from mice fed HFD +/− FEN treatment. HFD led to an accumulation of numerous acylcarnitines, indicative of impaired β-oxidation (Table 3). FEN completely normalised all of these elevations to levels measured in CHOW-fed mice (Table 3). FEN also completely prevented the rise in tricarboxylic acid (TCA)-cycle intermediates and oxidative stress markers (Table 3). This strongly suggests that FEN-treatment *in vivo* can alleviate disturbances to adipose tissue mitochondrial function resulting from HFD feeding.

### 3.7 FEN can induce markers of cellular stress and autophagy in mature adipocytes

**Fig. 6:**
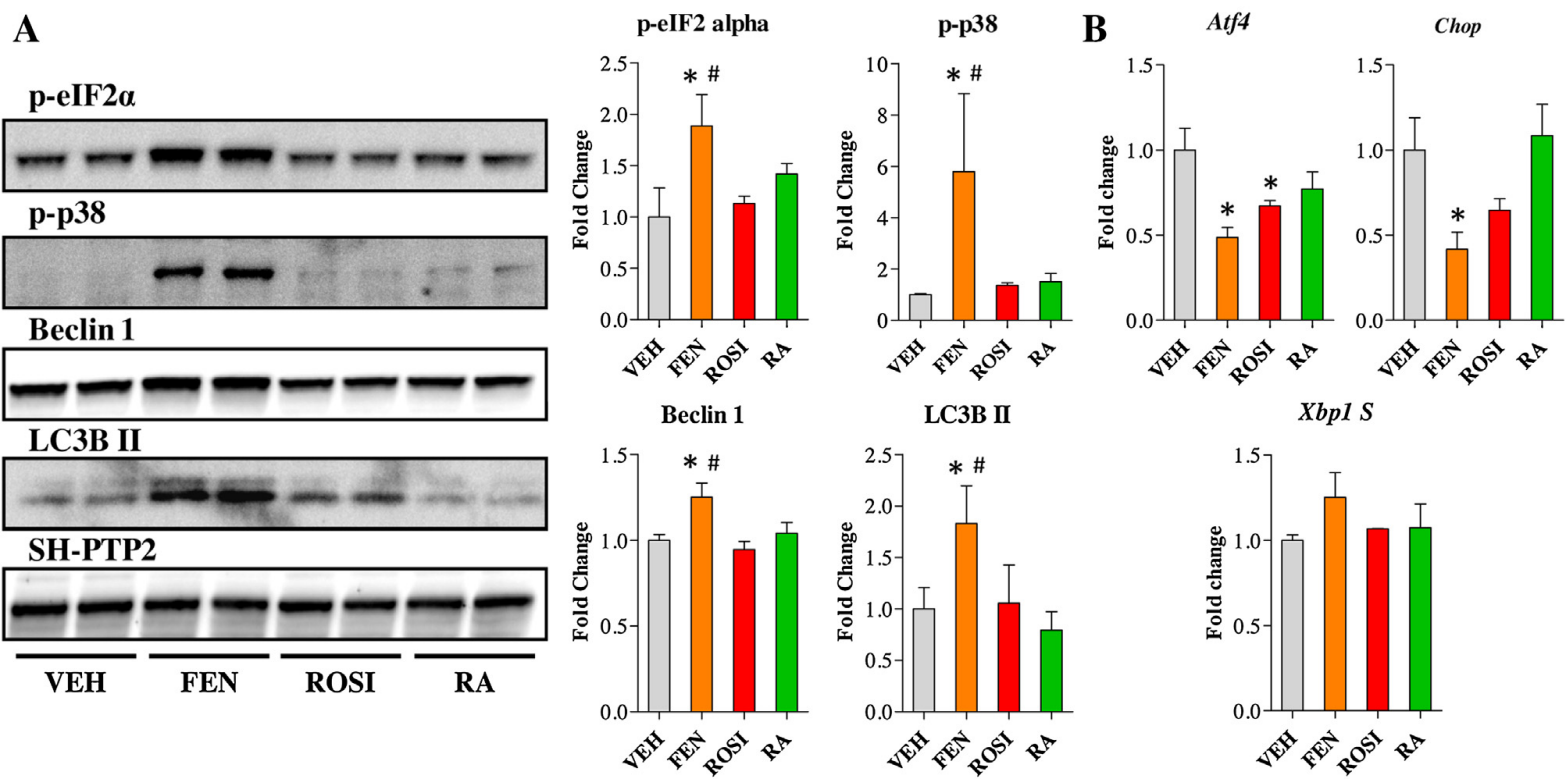
FEN induces markers of cellular stress and autophagy in 3T3-L1 adipocytes. (A) Western blot analysis of ER stress and autophagy markers in 3T3-L1 adipocytes differentiated for 16 days with indicated compounds added from day 8. Right panels are quantification of western blots in left panels. Proteins were normalised to SH-PTP2 levels, *n* = 4. Significance *P < 0.01 vs VEH and #*P* < 0.05 vs RA. (B) Gene expression analysis of ER stress markers at day 16 of differentiation. Significance *P < 0.01 vs VEH, *n* = 3.

To determine whether FEN’s inhibition of ceramide biosynthesis was associated with the induction of cellular stress and survival responses as reported in cancer cell lines, we examined the levels of phospho-eIF2α, phospho-p38 and markers of autophagy. FEN increased levels of phospho-eIF2α (downstream of the PERK stress response pathway) and phospho-p38 (a mitogen activated protein kinase) (Fig. 6a). FEN also increased protein expression of Beclin1 and LC3B II which are required for autophagosome formation (Fig. 6a). FEN did not increase the expression of *Atf4*, the spliced isoform of *Xbp1* or *Chop.* Surprisingly, FEN decreased gene expression levels of *Atf4* and *Chop*, which may indicate the induction of pro-survival pathways rather than progression to apoptosis in 3T3-L1 cells (Fig. 6b).

### 3.8 FEN metabolite 4-OXO cannot inhibit 3T3-L1 adipogenesis

**Fig. 7:**
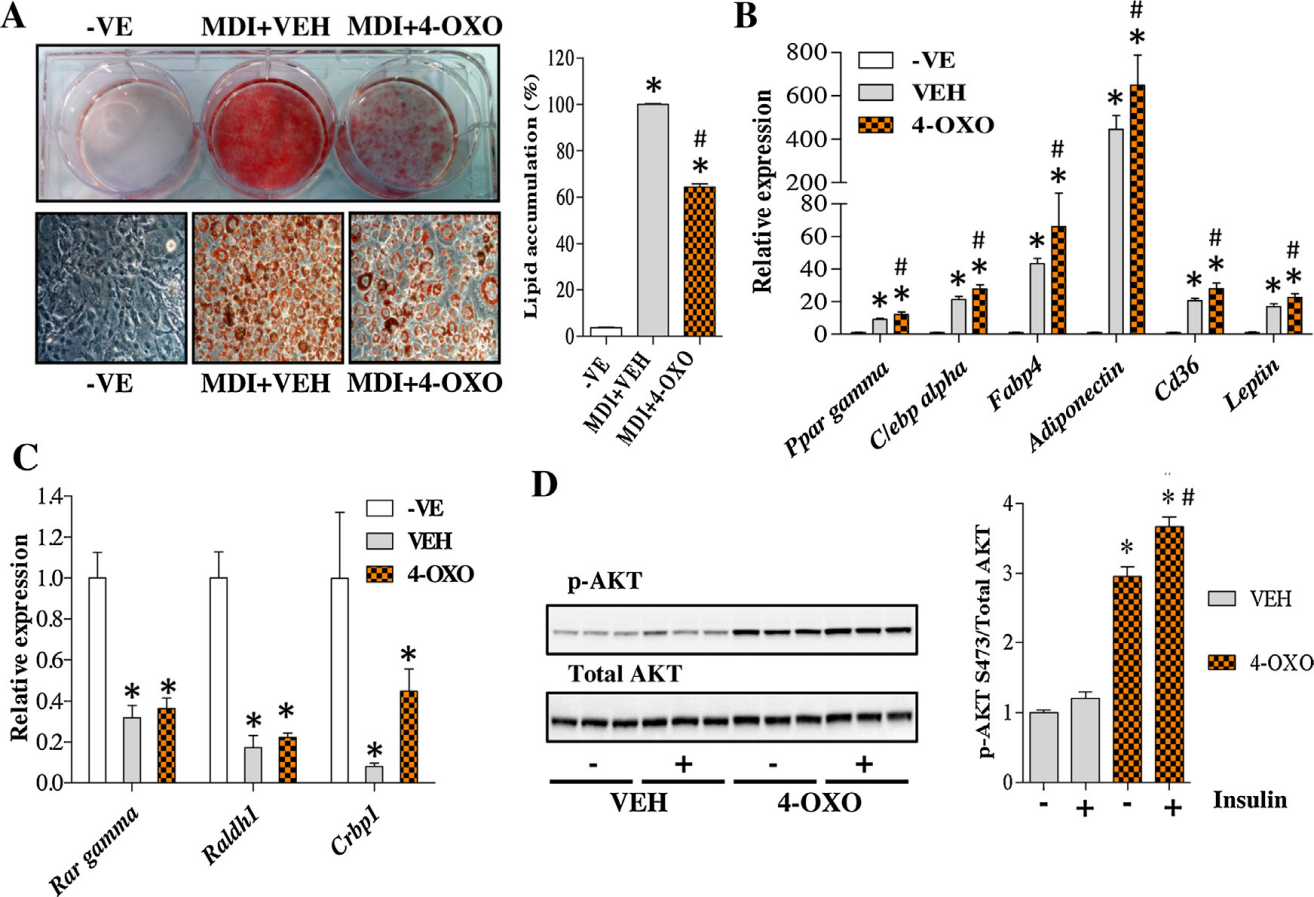
FEN catabolite 4-OXO does not inhibit 3T3-L1 adipogenesis. (A) Lipid stained 3T3-L1 adipocytes differentiated with indicated compounds. Bottom left panels are 200× images of cell morphology from upper left panel. Right panel is quantification of lipids. Significance *P < 0.001 vs -VE and #*P* < 0.001 vs VEH, *n* = 4. Gene expression analysis of adipogenic (B) or retinoid markers (C). Significance *P < 0.01 vs -VE and #*P* < 0.05 vs VEH. Expression data was normalised to *Nono*, *Ywhaz* and *18S*, *n* = 4. (D) Western blot analysis of p-AKT S473 in differentiated adipocytes. Cells were serum starved for 16hr and treated with 20 nM insulin (+) or saline (−) for 15 min. Right panel is quantification of western blots. Proteins were normalised to total AKT levels, *n* = 3. Significance *P < 0.001 vs VEH (−) and #*P* < 0.01 vs 4-OXO (−).

FEN can activate both RAR-dependent and -independent signalling pathways in 3T3-L1 cells. Previous studies have determined that 4-oxo-*N*-(4-hydroxyphenyl)retinamide, an oxidised FEN catabolite hereafter termed 4-OXO [41], operates in a RAR-independent manner, can manipulate ceramide biosynthesis similarly to FEN and is more potent than the parent compound [42], [43]. We tested whether 4-OXO was responsible for the inhibition of adipogenesis and/or decreased lipid accumulation in 3T3-L1 cells. 4-OXO was unable to inhibit adipogenesis in 3T3-L1 cells, however, lipid accumulation was less than in VEH treated cells (Fig. 7a). This effect was not due to toxicity as concentrations up to 10 μM were well tolerated (data not shown). Unexpectedly, 4-OXO resulted in increased expression levels of adipogenic markers (Fig. 7b). Retinoid genes were down regulated by differentiation and 4-OXO was unable to induce any of these genes above the levels observed in VEH (Fig. 7c). Thus, the catabolism of FEN to 4-OXO does not contribute to the inhibition of 3T3-L1 differentiation, however it appears 4-OXO may have non-retinoid, insulin-sensitizing properties. To test this further, insulin stimulated phosphorylation of Akt (phospho-Akt) was examined. VEH treated cultures responded poorly to insulin stimulation with only small increases in phospho-Akt levels observed when compared to saline controls (Fig. 7d). This is likely to be a result of the development of insulin resistance through chronic insulin treatment, which has been described previously [44]. 4-OXO increased phospho-Akt levels by almost 3-fold compared to VEH controls in the absence of insulin. 4-OXO further increased phospho-Akt levels with insulin stimulation indicating that treatment with 4-OXO results in increased insulin signalling in differentiated 3T3-L1 adipocytes (Fig. 7d).

**Fig. 8:**
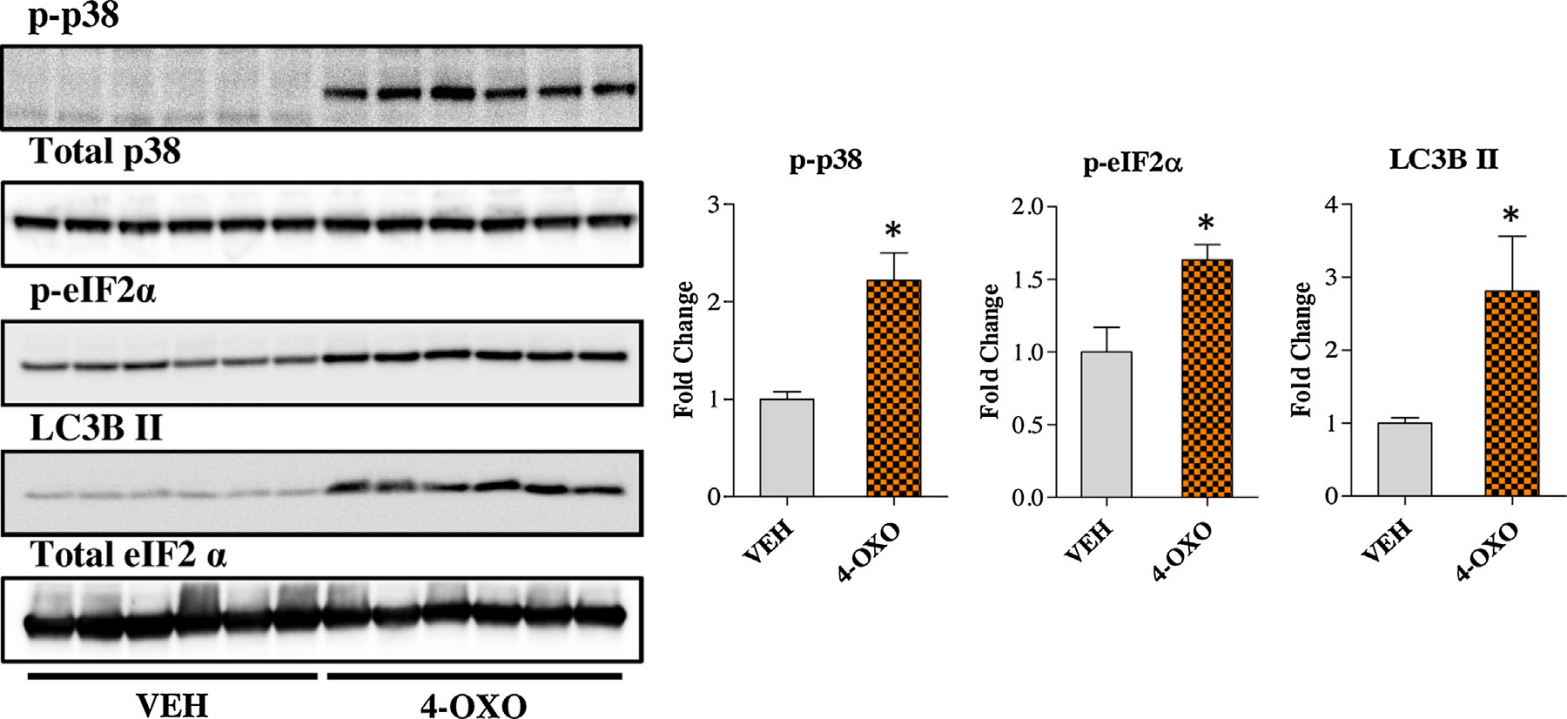
4-OXO induces cellular stress and autophagy markers. Western blot analysis of ER stress and autophagy markers in 3T3-L1 adipocytes differentiated with indicated compounds. Right panels are quantification of western blots in left panels. Proteins were normalised to total p38 or eIF2α levels, *n* = 6. Significance *P < 0.001 vs VEH by *t*-test.

Next we investigated whether 4-OXO exhibited other RA-independent signalling properties as observed with FEN treatment. 4-OXO led to increased levels of phospho-p38, phospho-eIF2 α and LC3B II indicating the induction of cellular stress responses and autophagy induction (Fig. 8). Thus, the RA-independent effects of FEN treatment are also exhibited by 4-OXO in 3T3-L1 adipocytes.

## 4 Discussion

The data presented here demonstrates that FEN can increase dihydroceramide and dihydrosphingomyelin lipid species independently of RAR-signalling, which is associated with activation of cellular stress responses and decreased lipid content in mature 3T3-L1 adipocytes. Moreover, our translational findings from mouse adipose tissue suggest that the manipulation of ceramide biosynthesis is also linked to normalisation of mitochondrial function in association with FEN-mediated inhibition of HFD-induced obesity and insulin resistance. This is in contrast to the mechanism of FEN action to inhibit 3T3-L1 differentiation, which we have demonstrated is mediated by ligand-induced activation of RARα signalling and induction of genes involved in retinoid metabolism (summarised in the graphical abstract).

Whereas RA was able to completely inhibit adipogenesis in the presence of ROSI, FEN was only able to partially prevent lipid accumulation. ROSI can stimulate adipogenesis *via* increased PPARγ occupancy at nearby target genes but can also repress some genes that appear to be more dependent on C/EBPα [45]. Our results suggest that the ROSI-induced down-regulation of retinoid homeostasis genes can be overcome by RA *via* co-ordinate inhibition of C/EBPα and early induction of retinoid responsive genes such as *Crbp1* and *Rar gamma*. Under these conditions, the less potent FEN could not repress C/EBPα or induce retinoid responsive genes and thus could not prevent adipogenesis. Although liganded RAR has been known to inhibit adipogenesis through the prevention of C/EBPβ mediated transcription, recent studies have also pointed to an inhibitory role for CRBP1 in adipogenesis and triacylglyceride accumulation [3].

The ability of FEN treatment to decrease adipocyte lipid content in mature adipocytes was found to be RARα independent, consistent with previous reports that RA is unable to replicate this effect [6], [27], [28]. Moreover, dihydroceramide lipid species increased specifically in FEN-treated adipocytes, similarly to reported effects in carcinoma cells [37], [46]. This effect appears to occur *via* direct inhibition of Des1 activity and mRNA, which catalyses the final step in *de novo* ceramide synthesis [43]. However, this is the first report that FEN can cause such alterations or in addition, inhibit *Cers6* gene expression in adipocytes or adipose tissue. Thus, the complete normalisation of impaired mitochondrial β-oxidation and TCA cycle flux in adipose tissue suggests that *in vivo*, FEN may inhibit obesity and insulin resistance *via* inhibition of ceramide-induced insulin resistance and attenuation of mitochondrial dysfunction.

Accumulation of numerous acylcarnitines and ceramide linked to excess lipid supply and impaired mitochondrial fatty acid β-oxidation in skeletal muscle is associated with muscle insulin resistance [47], [48]. Moreover, S. Summers and co-workers have shown FEN to directly prevent lipid induced insulin resistance in cultured myotubes and isolated muscles strips in association with increases in dihydroceramide levels [18]. However, insulin sensitization after treatment with the thiazolidinedione class of PPARγ agonists is associated with increased adipose mitochondrial capacity and expression of adipose genes essential for branched-chain amino-acid oxidation, fatty acid β-oxidation, TCA-cycle and oxidative phosphorylation pathways [49], [50]. Thus overall, FEN appears to correct these impairments in lipid homeostasis pathways in multiple insulin sensitive tissues *in vivo* [51], [52], [53], [54].

The FEN metabolite 4-OXO [41] has been identified as biologically active and inhibits Des1 more potently than FEN [43]. Thus, 4-OXO may be partly responsible for some of the effects observed in response to FEN treatment [42]. We have revealed that 4-OXO is poor at inducing RAR-signaling and thus unable to inhibit 3T3-L1 adipogenesis. However, 4-OXO increased adipogenic biomarkers and insulin sensitivity. Thus, elevated p-AKT levels are probably due to the non-RAR signaling characteristics of FEN and FEN-like compounds, such as inhibition of ceramide synthesis [37], [43]. These findings identify that 4-OXO may be a novel and previously unexplored therapeutic candidate to improve adipocyte insulin sensitivity without negatively impacting upon adipocyte differentiation or other non-beneficial effects of retinoid treatment.

We found that both FEN and 4-OXO, (but not RA), led to increases in markers of cellular stress and autophagy without inducing apoptotic cell death. Increased dihydroceramide species presents a potential non-RAR mechanism of FEN/4-OXO action linked to modulation of nutrient stress pathways. Regulation of these pathways has been shown to be impaired and contribute to the pathogenesis of diabetes [22], [23]. Induction of ER stress and autophagy has been observed in variety of different cells types after FEN or dihydroceramide supplementation [19], [20], [21] and may account for the decrease in adipocyte lipid content in mature 3T3-L1 adipocytes. A recent study indicated that hypertrophic adipocytes fail to process autophagasomes through reduced autophagic flux [55]. This suggests defective autophagy may be associated with or even responsible for increases in adiposity and states of obesity. FEN and 4-OXO may increase autophagic flux and alleviate cellular disturbances that arise in hypertrophic adipocytes as a result of diet-induced obesity. However, further investigations will be required to determine the importance of this finding *in vivo*.

Overall, our findings indicate that FEN appears to display the unique ability to inhibit adipocyte differentiation and hypertrophy *in vitro* and these effects are mediated through distinct mechanistic pathways that are translated *in vivo*. We have confirmed that like RA, FEN inhibits adipocyte differentiation *in vitro* through RARα-dependent signaling. However, the non-RAR effects of FEN and its catabolite 4-OXO to modulate ceramide synthesis appear to be linked to improved mitochondrial function and modulation of cellular stress responses and autophagy. Future experiments will be required to demonstrate a causal molecular link between the alterations in ceramide synthesis and the observed metabolic outcomes. Our findings suggest that this unique combination of biological effects may be responsible for the low toxicity and beneficial effects of FEN treatment to inhibit obesity and insulin resistance.

## Conflict of interest

No potential conflicts of interest relevant to this article were reported.

## Author’s contribution

GDM and NM made contributions to all areas of the submitted work including study conception and design, acquisition, analysis and interpretation of data and drafting/revision of the work for intellectual content and context. SRT, BHM, LG, MKD, DGW, MD and PDW contributed to acquisition, analysis and/or interpretation of data. MD also contributed the study conception and design. NM takes overall responsibility for the work including final approval.

## Figures and Tables

**Table 1:** Identification of the major lipid species altered with Fenretinide treatment in mature 3T3-L1 adipocytes. LC–MS analysis was performed on lipid extracts from 3T3-L1 adipocytes differentiated for 16 days. FEN and RA at 1 μM or equivalent DMSO as VEH added at day 8, *n* = 7 for VEH, *n* = 8 for FEN and RA. Significance was calculated by one-way ANOVA, **P < 0.01 and ***P < 0.001.

Increased specifically with FEN (fold change)					Decreased specifically with FEN (fold change)				
Lipid ID	vs VEH	*P* value	vs RA	*P* value	Lipid ID	vs VEH	*P* value	vs RA	*P* value
Cer (34:0)			5.27	***	SM (34:1)			2.23	***
Cer (40:0)	8.63	***			SM (42:3)	2.19	***		
Cer (42:0)	16.45	***							
					PE (34:0)			2.21	***
SM (33:0)	70.01	***	21.03	***	PE (34:1)			3.64	***
SM (34:0)	4.32	***	3.93	***	PE (36:1)			2.57	***
					PE (36:2)			4.47	***
PC (38:4)			2.50	***					
					PC (32:1)	4.02	***	4.14	***
DG (34:0)	1.76	***			PC (32:2)	6.66	***	8.50	***
DG (36:3)			1.32	**	PC (34:1)			1.97	***
DG (38:3)	4.30	***			PC (34:2)			2.78	***
DG (O-34:1)	3.23	***	2.44	***					
					TG (36:0)	6.71	***	2.74	***
TG (48:4)	32.25	***	46.10	***	TG (37:0)	8.87	***	3.28	***
TG (56:4)			1.31	**	TG (38:0)	6.12	***		
	TG (38:1)	10.79	***	3.94	***				
	TG (39:1)	6.62	***	2.90	***				
					TG (40:0)	4.82	***		
					TG (40:1)	7.76	***	2.50	***
					TG (40:2)	9.53	***	3.48	***
					TG (42:2)	4.97	***	2.16	***
					TG (44:0)	1.93	***		
					TG (44:2)	3.00	***		
					TG (45:2)	2.44	***		
					TG (46:2)	2.73	***		
					TG (46:3)	3.42	***	2.08	***
					TG (47:3)	3.09	***		
					TG (48:2)			1.50	***
					TG (49:2)	2.12	***		
					TG (50:4)	2.73	***		
					TG (51:3)	5.44	***	3.04	***
					TG (53:3)			1.82	***

Key: Cer = ceramide; SM = sphingomyelin; PE = phosphatidylethanolamine; PC = phosphatidylcholine; PS = phosphatidylserine; TG = triglyceride; DG = diglyceride.

**Table 2:** Identification of the major lipid species increased or decreased by both FEN and RA or specifically RA treatment in mature 3T3-L1 adipocytes. LC–MS analysis was performed on lipid extracts from 3T3-L1 adipocytes differentiated for 16 days. FEN and RA at 1 μM or equivalent DMSO as VEH added at day 8, *n* = 7 for VEH, *n* = 8 for FEN and RA. Significance was calculated by one-way ANOVA, **P < 0.01 and ***P < 0.001.

Increased by both FEN and RA (fold change vs VEH)				
Lipid ID	FEN	*P* value	RA	*P* value
PC (44:3)	9.54	***	7.59	***
PS (44:1)	4.00	***	3.45	***
PS (44:2)	6.20	***	5.03	***
TG (56:6)	2.33	***	2.55	***
TG (56:7)	3.11	***	3.87	***
TG (57:4)	3.18	***	2.98	***
TG (57:5)	14.12	***	12.18	***
TG (57:6)	17.74	***	17.76	***
TG (58:7)	11.00	***	14.39	***

Increased specifically by RA (fold change vs VEH)				
Lipid ID	FEN	*P* value	RA	*P* value
PS (44:3)	2.51			***

Decreased by both FEN and RA (fold change vs VEH)				
Lipid ID	FEN	*P* value	RA	*P* value
TG (38:1)	10.79	***	2.74	***
TG (40:1)	7.76	***	3.10	***
TG (44:1)	2.85	***	2.09	***
TG (45:1)	2.41	***	1.88	***
TG (46:1)	2.51	***	1.95	***
TG (47:1)	2.05	***	1.75	***
TG (48:1)	2.22	***	1.86	***
TG (48:2)	3.08	***	1.76	***
TG (48:5)	2.73	***	1.66	***
TG (49:1)	1.62	***	1.58	***
TG (50:1)	1.43	***	1.60	***
TG (50:2)	1.95	***	1.46	***
TG (51:2)	2.97	***	1.68	***

Decreased specifically by RA (fold change vs VEH)				
Lipid ID	FEN	*P* value	RA	*P* value
TG (46:0)			1.60	***
TG (47:0)			1.39	***
TG (48:0)			1.40	**
TG (49:0)			1.39	***
TG (52:1)			1.35	***

**Table 3:** Metabolomics analysis of PG-WAT from HFD mice treated with FEN. Levels of carnitine, TCA cycle intermediates and oxidative stress markers in PG-WAT following global metabolite analysis by LC–MS. For each group *n* = 5 mice. All data represents mean and significance **P < 0.01 and ***P < 0.001 vs CHOW by one-way ANOVA with Tukey post hoc test.

*m*/*z*	Retention time	Compound	Acyl chain	Relative to CHOW	
				HFD	FEN-HFD
Carnitines					
162.113	12.9	(*S*)-Carnitine		0.40***	0.53***
248.149	11.0	Hydroxybutyrylcarnitine	C4:0	2.91**	0.85
276.180	8.8	Hydroxyhexanoylcarnitine	OH–C6:0	3.86**	1.21
342.264	4.6	*trans*-2-Dodecenoylcarnitine	C12:1	2.89**	1.08
344.279	4.6	Dodecanoylcarnitine	C12:0	3.18**	0.88
360.275	4.6	2-Hydroxylauroylcarnitine	OH–C12	8.77**	2.03
370.295	4.6	*trans*-2-Tetradecenoylcarnitine	C14:1	3.14**	1.19
372.311	4.5	Tetradecanoylcarnitine	C14:0	3.68***	1.08
386.290	4.6	3-Hydroxy-*cis*-5-tetradecenoylcarnitine	OH–C14:1	11.52**	2.56
388.306	4.6	2-Hydroxymyristoylcarnitine	OH–C14:0	12.56**	1.91
398.327	4.5	9-Hexadecenoylcarnitine	C16:1	3.95**	1.01
400.342	4.4	[FA] *O*-Palmitoyl-*R*-carnitine	C16:0	5.31**	1.21
414.322	4.6	3-Hydroxy-9-hexadecenoylcarnitine	OH–C16:1	13.93**	4.16
416.337	4.6	2-Hydroxyhexadecanoylcarnitine	OH–C16:0	18.07***	2.53
426.358	4.3	11*Z*-Octadecenylcarnitine	C18:1	8.55**	1.69
442.353	4.5	3-Hydroxy-9*Z*-octadecenoylcarnitine	OH–C18:1	22.25***	3.26

TCA intermediates					
173.010	17.5	Aconitate		1.83***	1.02
147.030	13.1	2-Hydroxyglutaric acid		2.30**	0.8
145.014	14.9	Ketoglutaric acid		6.07**	0.80
133.015	15.4	Malic acid		1.80***	0.94
115.004	15.4	Fumaric acid		1.87***	0.95
117.019	14.5	Succinic acid		1.48**	0.86
191.020	17.7	Citrate		2.73**	0.89

Oxidative stress					
308.091	13.5	Glutathione		2.05**	0.88
613.160	16.9	GSSG		1.60	0.78
